# Parental death and parental substance use problems during childhood and the risk of DSM-5 substance use, suicidality and mental health disorders

**DOI:** 10.1007/s00127-026-03076-6

**Published:** 2026-03-13

**Authors:** Christopher W. Giang, Rebecca J. Evans-Polce, Luisa Kcomt, Kara Dickinson, Holly Gurnik, Joshua Truchan, Issac Hess, Sean Esteban McCabe

**Affiliations:** 1 Center for the Study of Drugs, Alcohol, Smoking and Health, School of Nursing, University of Michigan, 400 N. Ingalls St, Ann Arbor, MI 48109 USA; 2 Department of Health Behavior and Health Equity, School of Public Health, University of Michigan, Ann Arbor, MI USA; 3 School of Social Work, Wayne State University, Detroit, MI USA; 4 Boston University School of Public Health, Boston, MA USA

**Keywords:** Parental death, Adverse childhood experiences, Suicide, Parental substance use, Substance use disorder, Mental health disorder

## Abstract

**Purpose:**

This study examines the associations between childhood parental death and parental substance-related problems and DSM-5 substance use disorders (SUDs), suicide attempt, and mental health discorders (mood, anxiety, post truamatic stress) in adulthood..

**Methods:**

Using data from the National Epidemiologic Survey of Alcohol and Related Conditions-III (*n* = 36,309), we compared four mutually exclusive groups who experienced the following before age 18: parental death and no parental substance use problems; parental substance use problems and no parental death; both parental death and parental substance use problems; and neither parental death nor substance use problems. We examined differences in adverse childhood experiences (ACEs), suicidality, and six DSM-5 SUD and mental health disorders. Parental death and parental substance-related problems were treated as exposures rather than included in the ACEs measure.

**Results:**

Parentally-bereaved individuals who experienced parental substance-related problems reported significantly greater number of ACEs (M = 5.34) compared to all other groups. Parentally-bereaved individuals who experienced parental substance-related problems had greater odds of a suicide attempt and all six DSM-5 SUD and mental health disorder outcomes (aOR range = 2.06–3.59) compared to parentally-bereaved individuals without parental substance-related problems. They also had greater odds of a suicide attempt and four DSM-5 SUD and mental health disorders (aOR range = 1.19–1.46) compared to those who experienced parental substance use problems and no parental death. Some differences were attenuated in models adjusting for ACEs; however, differences remained for six of the seven outcomes.

**Conclusion:**

Individuals who experienced parental death and parental substance-related problems have increased risk for suicidality, DSM-5 SUD, and mental health disorders which may warrant additional trauma-informed mental health care in bereavement services.

**Supplementary Information:**

The online version contains supplementary material available at https://doi.org/10.1007/s00127-026-03076-6.

## Introduction

Childhood bereavement in the United States (U.S.) reached a peak of over two million parentally-bereaved children in 2021 [1–3]. Approximately 2.9 million U.S. children (4.2%) experienced parental or primary caregiver death between 2000 and 2021, driven by rising deaths from drug overdose, suicide, and injury, with disproportionate impacts on adolescents and socioeconomically disadvantaged groups [4]. Experiencing the death of a parent or significant caregiver during childhood can have a substantial impact on a child’s emotional, behavioral, and mental well-being [5–6]. Individuals who had adverse childhood experiences (ACEs) have an increased risk of mental health and substance use disorders (SUDs) [7–11]. From 2011 to 2021, over 320,000 children lost a parent to drug overdose. Despite this, the combined impact of parental death and parental substance use on children’s later mental health, including suicidality, SUDs, and mental health disorders based on the Diagnostic and Statistical Manual of Mental Disorders, Fifth Edition (DSM-5) remains understudied.

Parental death is associated with an increased risk of developing mental health disorders for bereaved children, with evidence demonstrating heightened risk both in the period immediately following the death and across subsequent years [12]. This includes an increased risk of any mood disorders [13–16], particularly depressive disorders [15, 17–19]. Parentally-bereaved children have increased risk of bipolar disorder [20], though this seems to be concentrated in children whose parents died by suicide [21–22]. Anxiety disorders are also elevated for children who experienced a parental death [23–24], as is posttraumatic stress disorder (PTSD) [22, 25–27] and suicidal ideation [24, 28]. In addition, parentally-bereaved children have an increased risk of early substance use onset relative to their nonbereaved peers [29–31], suggesting the risk for DSM-5 SUDs among parentally-bereaved individuals in adulthood warrants more attention. Evidence from prior waves of the National Epidemiologic Survey on Alcohol and Related Conditions (NESARC) further demonstrates that the unexpected death of a loved one is associated with an elevated risk for the onset of mood, anxiety, and SUDs across the life course [32]. However, this work did not examine parental death in the context of parental substance use problems or broader patterns of ACEs, highlighting an important gap addressed by the present study.

In some cases, a parent’s death intersects with the adverse experience of having a parent or caregiver with substance-related problems. Parallel literature demonstrates substantial adverse consequences for children who are exposed to parental SUD. Between 2009 and 2014, approximately 8.7 million children in the U.S. lived with at least one parent with a diagnosed SUD based on DSM-IV criteria while more recent studies place this estimate closer to nearly 19 million children residing with a parent with a DSM-5 SUD [33]. Notably, nationally representative analyses indicate that the shift from DSM-IV to DSM-5 criteria is associated with an approximately 80% increase in the estimated number of youth exposed to parental SUD [34]. Recent nationally representative data using DSM-5 criteria indicate that nearly one quarter of U.S. children lived with at least one parent meeting criteria for SUD in 2023, highlighting the scope of children potentially affected by parental substance-related problems [35]. SUDs can impair an individual’s parenting ability and increase the risk of household dysfunction, neglect, material deprivation, and physical violence [36 − 27]. Children who have experienced parental SUD have an increased risk of mood and anxiety disorders [29, 36–37], PTSD [38], and SUDs [37, 39–40]. Living with parental SUD can create unsafe and unstable households for children and addressing these issues requires comprehensive support systems to ensure healthier environments. Evidence linking parental death to later substance use outcomes among offspring has been mixed, with a recent systematic review and meta-analysis and subsequent register-based study reporting substantial heterogeneity across studies and emphasizing the influence of study design, timing of exposure, and family context [41–42]. Together, this mixed evidence underscores the need to examine parental death and parental substance-related problems jointly, rather than in isolation.

Parental SUD intersects with suicide bereavement, as many parents who die by suicide have higher rates of SUD compared to parents who die by natural causes [21]. Children who experience parental suicide and SUD-related deaths face unique challenges, including societal stigma and increased vulnerability to suicidal thoughts, substance use, depression, anxiety, and PTSD [12, 15, 43–45]. Parental suicide may be more distressing than other types of parental deaths, though evidence is mixed. However, suicide and drug overdose deaths frequently carry societal stigma, distinguishing them from other causes of death [46–47]. Children and adult survivors of suicide and drug-related deaths often report feeling shamed, blamed, judged, and uncomfortable discussing the death [46, 48]. Surviving parents and relatives may conceal the cause of death due to stigma, which can limit support and increase a child’s sense of isolation, alienating them from peers [48–49]. Higher perceived stigma in suicide- and drug-related bereavement is associated with greater psychological distress, depression, self-harm, and suicidality [46, 50]. Without further research and tailored interventions, the combination of suicide bereavement, SUD, and societal stigma may have lasting impacts on children’s mental health and their willingness to seek support, complicating the grieving process.

Limited research has examined the intersection of parental death and parental substance-related problems, and much of the literature examines these specific adverse childhood experiences separately or do not control for other ACEs [1, 51]. Prior research indicates that the exact circumstances of the parent’s death can influence the severity of parentally-bereaved children’s mental health symptoms [15, 52]. Understanding the potential mental health outcomes for those who experience both parental death and parental substance-related problems is a current gap in the literature. Reasons mental health outcomes in adulthood may differ for this unique population include the sudden nature of the death [15, 22, 24, 52–54], greater likelihood of exposure to additional ACEs and household dysfunction in the family [55–56], stigma associated with drug overdose death [48, 57–59], and a greater predisposition for SUD due to a combination of genetic and familial environmental factors [39, 60–62]. In addition to genetic vulnerability, associations between parental substance use problems and adverse mental health outcomes among offspring may reflect shared familial and environmental factors, including parental mental illness [63], socioeconomic disadvantage [64], and residential instability [65].

This study examined the risk of DSM-5 mental health disorders (anxiety, mood, and PTSD), SUDs (alcohol, tobacco, and other drug use disorders), and suicide attempts among four groups of individuals based on their childhood experiences: (a) both parental death and substance-related problems, (b) parental death only, (c) parental substance-related problems only, and (d) neither, to inform targeted prevention and resource allocation. In this context, additional ACEs may reflect broader correlated adversity and shared familial context that could confound associations between parental death or parental substance use problems and later outcomes. ACEs may also plausibly lie on pathways linking these childhood experiences to adult disorders, however, the present study does not test mediation, and ACE-adjusted models are intended to contextualize associations rather than establish causal mechanisms. As a secondary focus, we also compared individuals who experienced parental death and parental substance-related problems as well as a parental/other adult (OA) suicide to those who experienced parental death and parental substance-related problems and no parental/OA suicide.

## Methods

### Study design

Data for this study came from the National Epidemiologic Study of Alcohol and Related Conditions-III (NESARC-III; *n* = 36,309 non-institutionalized U.S. civilians aged ≥ 18 years) using computer-assisted personal interviewing (CAPI) methods. Respondents were selected using a multistage probability sampling method, which included primary sampling units (PSUs) consisting of individual counties or groups of contiguous counties, secondary sampling units (SSUs) composed of clusters of Census-defined blocks, and tertiary sampling units formed by households within the SSUs, with oversampling of Black, Asian, and Hispanic respondents. The NESARC-III was conducted through in-person household interviews from April 2012 to June 2013 with a response rate of 60.1% and used a multistage, complex survey design with stratification and clustering [66]. The survey protocols were approved by the Institutional Review Boards of the National Institutes of Health, the National Institute on Alcohol Abuse and Alcoholism, and Westat. The NESARC-III is the only national survey that has a comprehensive battery of ACEs questions, family history of substance-related problems, and DSM-5 SUD and mental health disorder questions, including the Alcohol Use Disorder and Associated Disabilities Interview Schedule-5 (AUDADIS-5), a structured interview that assesses DSM-5 diagnostic criteria and can be administered by non-clinician interviewers [67]. Diagnoses derived from AUDADIS-5 represent standardized DSM-5-based research classifications and do not constitute clinical diagnoses made by licensed clinicians in real-world settings. More detailed information on the methodology for weighting, sample selection, and other information can be found elsewhere [66].

### Measures

*Parental death* was assessed using the following questions: “Did either of your (biological/adoptive) parents die before you were 18?” and “Did your stepparent die before you were 18?”

*Parental substance use problems* were assessed by asking: “Before you were 18 years old, was a parent or other adult living in your home a problem drinker or alcoholic?,” and “Before you were 18 years old, did a parent or other adult living in your home have some similar problems with drugs?”

*Adverse childhood experiences (ACEs)* were adapted from validated scales including the Childhood Trauma Questionnaire [68–69], the Conflict Tactics Scale [70], childhood sexual abuse questionnaire [71], and the Adverse Childhood Events study [72–73]. Participants were asked 21 items about their life experiences before the age of 18 [74] regarding psychological abuse, physical abuse, physical neglect, emotional neglect, childhood sexual abuse, among others. Responses were summed to create a scale of 0–21 with a higher score signifying a greater number of ACEs. The ACEs scales have demonstrated excellent reliability [74]. Parental death, parental substance-related problems, and parental/OA suicide were not included in our ACE measure because they were used as exposure variables. All childhood exposures, including parental death, parental substance-related problems, and other ACEs, were assessed retrospectively for experiences occurring before age 18, whereas SUDs, mental health disorders, and suicide attempts reflect lifetime DSM-5 outcomes assessed at the time of the adult interview.

*Lifetime SUDs* were assessed using the AUDADIS-5. Individuals who reported at least 2 of the 11 SUD symptom criteria within any 12-month period were categorized as having a lifetime SUD. DSM-5 *alcohol use disorder (AUD)* and DSM-5 *tobacco use disorder (TUD*) were examined as separate dichotomous outcomes. Questions about cocaine, sedatives, stimulants, heroin, marijuana, hallucinogens, club drugs, opioids, or other drugs were combined to create a dichotomous measure of DSM-5 *other drug use disorder (DUD).* This composite measure was used to ensure adequate statistical power for less prevalent drug classes and is consistent with prior NESARC-based studies [75]. Consistent with NESARC-III AUDADIS-5 scoring, DSM-5 criteria were applied separately for each substance, and the DUD indicator reflects meeting criteria for at least one non-alcohol, non-tobacco drug use disorder rather than aggregating symptoms across substances.

*Lifetime suicide attempt* was assessed using the question, “In your entire life did you ever attempt suicide?”

*Lifetime mental health disorders* were assessed using the AUDADIS-5 and included *anxiety disorders* (i.e., agoraphobia, generalized anxiety disorder, panic, social and specific phobias), *mood disorders* (i.e., bipolar, dysthymia, major depressive disorder), and *PTSD* [67, 76]. These disorders were grouped to reflect DSM-5 diagnostic domains, align with prior epidemiologic studies using NESARC-III data that examine broad psychiatric categories, and improve statistical power while capturing clinically meaningful patterns of psychiatric risk.

*Parental/OA suicide* was assessed using the question, “BEFORE you were 18 years old, did a parent or other adult living in your home commit suicide?”

Other covariates included *sex* (male or female), *race-ethnicity* (White, Black, Asian/Native Hawaiian/Other Pacific Islander, American Indian/Alaska Native, Hispanic), *age* (centered), *education* (less than college degree, college degree or higher), *sexual identity* (heterosexual/straight, lesbian/gay, bisexual), *U.S. region* (Midwest, Northeast, South, West), and *urbanicity* (urban or rural). Weighted descriptive characteristics of the analytic sample are presented in Table 1.

**Table 1:** Weighted descriptive characteristics (*n* = 36,309)

	*N* (%_weighted_) or M (SE)
Parental death and parental substance use problem	
Neither	24,152 (68.84%)
Parental death + NO parental substance use problem	2,486 (6.41%)
NO parental death + parental substance use problem	7,873 (22.11%)
Parental death + parental substance use problem	974 (2.63%)
*Sex*	
Male	15,862 (48.09%)
Female	20,447 (51.91%)
Age (years; range 18–90)	46.54 (0.19)
*Education*	
Less than college degree	27,394 (71.85%)
College degree or higher	8,915 (28.15%)
*Race-ethnicity*	
White	19,194 (66.19%)
Black	7,766 (11.79%)
American Indian/Alaska Native	511 (1.56%)
Asian/Native Hawaiian/Other Pacific Islander	1,801 (5.73%)
Hispanic	7,037 (14.74%)
*Sexual identity*	
Heterosexual/straight	34,644 (97.17%)
Gay/lesbian	586 (1.48%)
Bisexual	566 (1.35%)
*U.S. Region*	
Northeast	5,180 (18.24%)
Midwest	7,566 (21.48%)
South	14,532 (37.05%)
West	9,031 (23.23%)
*Urbanicity*	
Urban	30,193 (78.75%)
Rural	6,116 (21.25%)
Other adverse childhood events (range 0–21)	2.43 (0.03)
DSM-5 lifetime alcohol use disorder	10,001 (29.09%)
DSM-5 lifetime drug use disorder	3,548 (9.90%)
DSM-5 lifetime tobacco use disorder	9,747 (27.87%)
Suicide attempt	1,995 (5.16%)
DSM-5 lifetime mood disorder	8,642 (23.91%)
DSM-5 lifetime anxiety disorder	5,991 (16.96%)
DSM-5 lifetime post-traumatic stress disorder	2,339 (6.12%)

### Data analysis

We first estimated the prevalence of experiencing parental death alone, parental substance-related problems alone, experiencing both parental death and substance-related problems, or neither of these experiences. Next, we compared the mean number of ACEs for individuals in each of these mutually exclusive groups. We then conducted two sets of logistic regression models. The first set of models estimated the association of parental death and parental substance-related problems with lifetime suicide attempt, three lifetime SUD outcomes, and three lifetime mental health disorder outcomes, adjusting for sociodemographic characteristics. We estimated this association using those experiencing a parental death without parental substance use problems as the reference group and using those experiencing a parental substance use problem without parental death as the reference group. The second set of models estimated these associations while adjusting for sociodemographic characteristics and other ACEs. We present findings from both sets of models because other ACEs may play a key mediating or confounding role in these associations. Finally, in supplemental models, we estimated associations of parental death, parental substance-related problems, and parental/OA suicide with lifetime SUDs, suicide attempt, and mental health disorders adjusting for relevant covariates. All analyses used the svy command in Stata to adjust for nonresponse and the sample design. Participants with missing data on exposures, outcomes, or covariates were excluded from the analytic sample. Missing data were minimal for primary study variables (< 5%).

## Results

One quarter of adults reported having a parent with a substance use problem (24.7%; See Table 1). Fewer experienced a parental death prior to age 18 (9.0%). Of those who experienced a parental death, 28.2% also experienced having a parent with a substance use problem; this represented 2.6% of adults overall. Individuals differed on the number of other ACEs they experienced based on their history of parental death and parental substance use problems (see Table 2). Individuals who reported both a parental death and a parental substance use problem experienced a significantly greater number of ACEs (M = 5.34[SE = 0.23] *p* < 0.05) compared to those who had experienced a parental death without a parental substance use problem (M = 1.83[SE = 0.08] *p* < 0.05), experienced a parental substance use problem without a parental death (M = 4.62[SE = 0.07] *p* < 0.05), or experienced neither (M = 1.57[SD = 0.03]).

**Table 2:** Weighted mean number of ACEs (0–21) by experiences of a parental death and parental substance use problem

	Mean (SE)
Parental death and parental substance use problem	
Neither	1.57 (0.03)
Parental death + NO parental substance use problem	1.83 (0.07)
NO parental death + parental substance use problem	4.62 (0.07)
Parental death + parental substance use problem	5.34 (0.23)^a^

^a^Indicates significantly greater than three other groups at *p* < 0.05 based on bivariate linear regression model using the Parental death + parental substance use problem group as the reference

As shown in Table 3, those who experienced both a parental death and a parental substance use problem had the greatest estimated prevalence of all seven outcomes compared to the three other subgroups. For example, lifetime DSM-5 TUD was most prevalent 46.52% (95% CI = 42.77, 50.31%) among individuals who experienced both a parental death and a parental substance use problem relative to the other subgroups (range: 23.52% − 39.04%).

**Table 3:** Prevalence of DSM-5 lifetime substance use disorders, suicide attempt, and mental health disorders as a function of parental death and parental substance use problem

	DSM-5 Alcohol Use Disorder	DSM-5 Other Drug Use Disorder	DSM-5 Tobacco Use Disorder	Suicide Attempt	DSM-5 Mood Disorder	DSM-5 Anxiety Disorder	DSM-5 PTSD	
Parental death and parental substance use problem	% (95% CI) *n*	% (95% CI) *n*	% (95% CI) *n*	% (95% CI) *n*	% (95% CI) *n*	% (95% CI) *n*	% (95% CI) *n*	
Neither	25.35% (24.36, 26.36%) *n* = 5,715	7.21% (6.70, 7.75%) *n* = 1,686	23.52% (22.55, 24.51%) *n* = 5,464	3.28% (3.01, 3.58%) *n* = 846	20.06% (19.30, 20.84%) *n* = 4,790	14.13% (13.44, 14.84%) *n* = 3,305	4.30% (3.96, 4.67%) *n* = 1,070	
Parental death + NO parental substance use problem	23.76% (21.72, 25.94%) *n* = 532	7.16% (5.94, 8.60%) *n* = 166	27.95% (25.79, 30.21%) *n* = 644	3.61% (2.79, 4.65%) *n* = 99	19.66% (17.81, 21.65%) *n* = 505	13.30% (11.58,15.24%) *n* = 316	4.98% (4.09, 6.05%) *n* = 122	
NO parental death + parental substance use problem	41.19% (39.61, 42.78%) *n* = 3,156	17.81% (16.65, 19.03%) *n* = 1,438	39.04% (37.24, 40.89%) *n* = 2,980	10.17% (9.21, 11.22%) *n* = 839	35.56% (33.97, 37.18%) *n* = 2,770	25.46% (24.17, 26.79%) *n* = 1,961	11.23% (10.22, 12.32%) *n* = 943	
BOTH parental death + parental substance use problem	42.10% (37.94, 46.37%) *n* = 398	21.53% (18.18, 25.31%) *n* = 182	46.52% (42.77, 50.31%) *n* = 407	13.31% (10.69, 16.46%) *n* = 135	37.57% (33.82, 41.47%) *n* = 377	28.69% (25.15, 32.52%) *n* = 274	13.39% (10.98, 16.23%) *n* = 142	

CI=confidence interval, PTSD=post-traumatic stress disorder. Alcohol use disorder, tobacco use disorder and other drug use disorder diagnoses reflect research-based classifications and not clinical diagnoses

As shown in Table 4, those who experienced both a parental death and a parental substance use problem had greater odds of all lifetime SUDs, mental health disorders, and suicide attempt (aOR range: 2.06–3.59, *p* < 0.05) compared to those who experienced a parental death and no parental substance use problem (see Models 1–7). Additionally, those who experienced a parental substance use problem and no parental death had greater odds of all lifetime SUD, mental health disorders, and suicide attempt (aOR range: 1.47–2.47, *p* < 0.05) compared to those who experienced a parental death and no parental substance use problem. When compared to those who experienced a parental substance use problem without a parental death (Table 5, Models 15–21), those who experienced both a parental death and a parental substance use problem had greater odds of lifetime DUD, SUD, suicide attempt, mood disorder, and anxiety disorder (aOR range: 1.19–1.46, *p* < 0.05).

**Table 4:** Associations of parental death and parental substance use problems with lifetime substance use disorders, suicide attempt, and mental health disorders adjusting for sociodemographic characteristics (models 1–7) and adverse childhood experiences (models 8–14)

	Lifetime AUD aOR (95% CI)	Lifetime DUD aOR (95% CI)	Lifetime TUD aOR (95% CI)	Lifetime Suicide Attempt aOR (95% CI)	Lifetime Mood Disorder aOR (95% CI)	Lifetime Anxiety Disorder aOR (95% CI)	Lifetime PTSD aOR (95% CI)
Parental death and parental substance use problem^a^ (Models 1–7)							
Neither	0.85 (0.76, 0.96)*	0.84 (0.66, 1.07)	0.75 (0.67, 0.84)*	0.84 (0.63, 1.10)	0.94 (0.83, 1.06)	1.02 (0.86, 1.21)	0.81 (0.65, 1.02)
Parental death + NO parental substance use problem	Reference	Reference	Reference	Reference	Reference	Reference	Reference
NO parental death + parental substance use problem	1.76 (1.54, 2.00)*	2.23 (1.80, 2.76)*	1.47 (1.29, 1.67)*	2.47 (1.89, 3.21)*	1.90 (1.66, 2.18)*	1.95 (1.62, 2.34)*	2.05 (1.64, 2.56)*
Parental death + parental substance use problem	2.06 (1.67, 2.55)*	3.11 (2.23, 4.32)*	2.08 (1.75, 2.48)*	3.59 (2.47, 5.22)*	2.26 (1.81, 2.81)*	2.41 (1.84, 3.15)*	2.63 (1.87, 3.72)*
Parental death and parental substance use problem with ACEs adjusted (Models 8–14)							
Neither	0.87 (0.77, 0.98)*	0.87 (0.68, 1.12)	0.76 (0.68, 0.85)*	0.89 (0.68, 1.18)	0.97 (0.85, 1.10)	1.06 (0.89, 1.26)	0.87 (0.70, 1.08)
Parental death + NO parental substance use problem	Reference	Reference	Reference	Reference	Reference	Reference	Reference
NO parental death + parental substance use problem	1.35 (1.18, 1.56)*	1.55 (1.24, 1.94)*	1.13 (0.99, 1.29)	1.28 (0.97, 1.68)	1.28 (1.13, 1.46)*	1.33 (1.09, 1.61)*	0.98 (0.79, 1.23)
Parental death + parental substance use problem	1.49 (1.19, 1.86)*	1.98 (1.41, 2.77)*	1.51 (1.26, 1.82)*	1.60 (1.07, 2.39)*	1.37 (1.10, 1.70)*	1.49 (1.14, 1.95)*	1.05 (0.74, 1.49)

aOR=adjusted odds ratio, CI=confidence interval, AUD=alcohol use disorder, DUD=drug use disorder, TUD=tobacco use disorder, PTSD=post-traumatic stress disorder. Models 1–7 adjusted for sex, age, race/ethnicity, education, sexual identity, region, and urbanicity. Models 8–14 were adjusted for variables in Models 1–7 and adverse childhood experiences (ACEs). Alcohol use disorder, tobacco use disorder and other drug use disorder diagnoses reflect research-based classifications and not clinical diagnoses

**Table 5:** Associations of parental death and parental substance use problems during childhood with lifetime substance use disorders, suicide attempt, and mental health disorders using no parental death and parental substance use problem as a reference group

	Lifetime AUD aOR (95% CI)	Lifetime DUD aOR (95% CI)	Lifetime TUD aOR (95% CI)	Lifetime Suicide Attempt aOR (95% CI)	Lifetime Mood Disorder aOR (95% CI)	Lifetime Anxiety Disorder aOR (95% CI)	Lifetime PTSD aOR (95% CI)
Parental death and parental substance use problem (Models 15–21)							
Neither	0.49 (0.45, 0.53)*	0.38 (0.34, 0.42)*	0.51 (0.47, 0.55)*	0.34 (0.30, 0.38)*	0.49 (0.46, 0.53)*	0.52 (0.48, 0.57)*	0.40 (0.36, 0.44)*
Parental death + NO parental substance use problem	0.57 (0.50, 0.65)*	0.45 (0.36, 0.56)*	0.68 (0.60, 0.77)*	0.41 (0.31, 0.53)*	0.53 (0.46, 0.60)*	0.51 (0.43, 0.62)*	0.49 (0.39, 0.61)*
NO parental death + parental substance use problem	Reference	Reference	Reference	Reference	Reference	Reference	Reference
Parental death + parental substance use problem	1.17 (0.98, 1.40)	1.39 (1.10, 1.77)*	1.42 (1.21, 1.65)*	1.46 (1.09, 1.95)*	1.19 (1.00, 1.40)*	1.24 (1.01, 1.52)*	1.29 (0.98, 1.69)

aOR=adjusted odds ratio, CI=confidence interval, AUD=alcohol use disorder, DUD=drug use disorder, TUD=tobacco use disorder, PTSD=post-traumatic stress disorder. All models adjusted for sex, age, race/ethnicity, education, sexual identity, region, urbanicity; *p < 0.05. Alcohol use disorder, tobacco use disorder and other drug use disorder diagnoses reflect research-based classifications and not clinical diagnoses

After adjusting for other ACEs, many associations were attenuated; however, some differences remained. Compared to those who experienced a parental death and no parental substance use problem (Table 4, Models 8–14), those who experienced both a parental death and a parental substance use problem had significantly greater odds of all lifetime SUD outcomes (aOR range: 1.49–1.98, *p* < 0.05), suicide attempt (aOR = 1.60, 95% CI = 1.07, 2.39), mood disorder (aOR = 1.37, 95% CI = 1.10, 1.70), and anxiety disorder (aOR = 1.49, 95% CI = 1.14, 1.95). Similarly, those who experienced a parental substance use problem and no parental death had greater odds of lifetime AUD (aOR = 1.35, 95% CI = 1.18, 1.56) and lifetime DUD (aOR = 1.55, 95% CI = 1.24, 1.94) and lifetime mood disorder (aOR = 1.28, 95% CI = 1.13, 1.46) and anxiety disorder (aOR = 1.33, 95% CI = 1.09, 1.61).

As a secondary focus we examined the potential role of parental/OA suicide during childhood. In unadjusted models (results not shown), individuals who experienced the combination of parental death, substance use problem, and parental/OA suicide had the highest prevalence of all outcomes when compared to the other subgroups. For example, 26.6% of those who experienced a parental death, a parental substance use problem, and a parental/OA suicide reported a lifetime suicide attempt of their own compared to the other subgroups (range: 3.2% − 12.1%). Similarly, 53.8% of individuals who experienced a parental death, a parental substance use problem, and a parental/OA suicide had a lifetime DSM-5 anxiety disorder compared to the other subgroups (range: 13.3% − 27.1%). Finally, 26.6% of individuals who experienced a parental death, a parental substance use problem, and a parental/OA had lifetime DSM-5 PTSD compared to the other subgroups (range: 4.3% − 12.6%). In multivariable regression models, individuals who experienced a parental death, a parental substance use problem, and a parental/OA suicide had significantly greater odds of suicide attempt (aOR = 3.18, 95% CI = 1.58, 6.42) and anxiety disorder (aOR = 2.51, 95% CI = 1.25, 5.06) compared to those who experienced a parental death, a parental substance use problem, and no parental/OA suicide (see Supplemental Table 1).

## Discussion

The findings of this study using a nationally representative sample show that 9.0% of individuals experienced a parental death as children, potentially representing over 30 million people in the U.S., including over 12 million who experienced both parental death and parental substance-related problems as children [77]. Findings from this study indicate high levels of other ACEs and an elevated risk for SUDs and mental health disorders, suggesting a need for more comprehensive bereavement and healthcare resources, especially for parentally-bereaved children who also experience parental substance-related problems.

Children who experienced both parental death and parental substance use problems before age 18 had significantly more ACEs than the other groups, heightening their risk for adverse downstream effects. Previous research has shown children who experience multiple ACEs may have increased risk of future mental health disorders, SUDs, and lower overall life satisfaction [78–79]. Additionally, children exposed to multiple ACEs are more likely to use substances as a coping mechanism [79]. Clinicians working with children who experience parental death in the context of parental substance use should prioritize systematic screening for parental substance use histories, co-occurring ACEs, and trauma-related symptoms, rather than assuming a uniform grief response. Evidence-based interventions such as the Family Bereavement Program [80] and Trauma and Grief Component Therapy (TGCT) [81] offer complementary frameworks for supporting bereaved children. The Family Bereavement Program emphasizes caregiver support, coping skills, and family functioning following loss, and the TGCT provides a trauma-informed approach when children exhibit traumatic stress responses. Importantly, not all parental deaths are experienced as traumatic and not all bereaved children are exposed to multiple ACEs. Accordingly, intervention selection and adaptation should be guided by assessed needs rather than cause of death alone. Across both approaches, a shared focus on malleable coping processes such as emotion regulation, meaning making, and caregiver child communication may be particularly relevant for reducing long term substance use and mental health risk. Future research is needed to identify which targetable coping mechanisms are most salient for children exposed to both parental death and parental substance use, and to adapt existing interventions to explicitly address parental substance use and suicide-related stressors among bereaved families.

Individuals who experienced both parental death and parental substance use problem before age 18 were more likely to meet criteria for a SUD, mood disorder, anxiety disorder, and report a suicide attempt than those who experienced neither parental death and parental substance use alone. Adjustment for other ACEs attenuated several associations, though the many differences persisted. This suggests that the cumulative burden of additional ACEs may contribute to increased SUD risk and should be considered in treatment for these children.

Notably, parental substance use problems in childhood, even in the absence of parental death, were strongly associated with many SUD and mental health outcomes. In several models, the magnitude of these associations was comparable to those observed among individuals who experienced both parental substance use problems and parental death. Importantly, the lower odds observed among individuals who experienced parental death without parental substance use problems in Table 4 does not indicate a protective effect of parental death. Rather, these comparisons likely reflect the exceptionally high level of risk conferred by parental substance use exposure, which is associated with chronic household instability, cumulative adversity, and shared genetic and environmental vulnerability. This pattern highlights parental substance use as a substantial and independent risk factor for adverse long-term outcomes and suggests that prevention strategies should broadly address parental substance use exposure during childhood. At the same time, the elevated risk observed among bereaved children with parental substance use problems may indicate additional vulnerability that warrants targeted support within this subgroup. Taken together, the attenuation of associations after adjustment for ACEs, alongside the persistence of elevated risk among individuals exposed to parental substance use problems both with and without parental death, suggests that parental substance use may represent a particularly potent and enduring source of risk. These patterns are consistent with the possibility that shared familial adversity and genetic vulnerability linked to parental substance use may play a central role in shaping long-term mental health outcomes, rather than parental death alone.

While the number of other childhood ACES accounted for many of the differences between parental death and parental substance use groups, some differences remained. Compared to those who experienced parental death alone, individuals who experienced both parental death and substance use problems had increased risk of all outcomes. Previous research shows children of parents with SUDs have an increased genetic predisposition to SUDs [37, 79] and mental health disorders [34]. Children learn to cope with the emotional hardships of parental SUD through various means that can either be beneficial or detrimental to their mental health [82]. Future research is needed to better understand additional mechanisms of increased risk among children experiencing both a parental death and parental substance use problem.

When considering the potential influence of parental/OA suicide, the odds of lifetime suicide attempt, anxiety disorder, and PTSD were significantly higher among individuals who experienced parental death, parental substance use problem, and parental/OA suicide relative to those who experienced parental death, parental substance use problem and no parental/OA suicide. These findings align with prior research showing elevated risk of suicidality [83], depression [14], and PTSD [38] among individuals bereaved by parental suicide, potentially due to shared genetic vulnerability, traumatic loss, and stigma-related barriers to support. Our results extend this literature by highlighting how co-occurring parental substance use problems may further intensify risk. However, it should be noted the sample size was smaller and confidence intervals larger for those who experienced the combination of parental death, substance use problem, and parental/OA suicide. This may suggest uncertainty in the estimated effect sizes, as well as high variability in the data so caution is warranted when interpreting these results. Future research with larger samples of those who experienced parental suicide and other stigmatized deaths is necessary to provide more precise estimates.

In recent years, researchers and health professionals examining parental bereavement have advocated for research, interventions, and policy changes to address the growing number of parentally-bereaved children in the U.S [84–86]. However, childhood bereavement is heterogeneous, and bereavement experiences and outcomes vary substantially depending on the cause and circumstances of parental death, the broader family environment, and co-occurring adversities. As a result, services for children who experience sudden parental death may need to be tailored, especially when parental death is accompanied by parental substance use problems [12, 87–90]. The number of U.S. children who have experienced a parental drug overdose death has increased in recent years to approximately 500,000 in the U.S. over the past two decades [1, 91]. Thus, it is critical to better understand and provide resources to address the needs of this growing population of parentally-bereaved children. Such resources may include trauma-informed mental health and bereavement services, screening and referral within pediatric and primary care settings, school-based supports, and efforts to improve access to services in bereavement service deserts, defined as geographic areas where children and families have little or no access to bereavement services [92]. To date, Michigan is the only U.S. state with population-level analyses documenting trends in parental death from stigmatized causes. In Michigan, both the number and proportion of children who lost a parent to drug overdose, suicide, homicide, or alcohol- and other drug-induced deaths increased markedly between 2000 and 2023, with these causes accounting for more than 40% of all parental deaths by 2023 [93]. Analyses of Michigan data also reveal substantial geographic inequities in bereavement care, with extensive bereavement service deserts identified in regions where parental mortality far exceeds available support services [92]. The lack of comparable analyses in other states limits understanding of national patterns and highlights the need for coordinated evidence-based responses that address both the emotional consequences of loss and the structural gaps in bereavement care.

Previous research suggests that early intervention following any parental death is beneficial and may result in decreased health risks [94]. Many interventions have been created to assist bereaved children in coping with parental death [95–99] (e.g. The Family Bereavement Program, Trauma-Focused Cognitive Behavioral Therapy for Childhood Traumatic Grief, and Trauma and Grief Component Therapy for Adolescents). However, bereavement interventions may more appropriately meet the needs of bereaved children by using more holistic approaches, targeting specific bereavement circumstances, and considering additional ACEs [99]. Additional resources beyond those directly addressing bereavement for children may be needed, particularly for those who have also experienced a parental substance use problem, given the high number of other ACEs they report experiencing.

A strength of this study includes the use of a large, nationally representative sample that allowed examination of distinct groups based on parental death and parental substance-related problems. The NESARC-III includes a comprehensive, standardized assessment of a wide range of ACEs alongside DSM-5 substance use and mental health disorders using the validated AUDADIS-5 instrument. This enabled us to examine how co-occurring childhood adversities shape associations between parental death, parental substance use, and adult psychiatric outcomes, an area that has been underexplored in prior work. Together, these features strengthen the study’s contribution to understanding the cumulative and overlapping nature of childhood adversity in relation to long-term mental health and substance use risk.

We note several study limitations. The NESARC-III is a cross-sectional study, which precludes a prospective examination of these associations across the life course from childhood to adulthood. Thus, temporal ordering between childhood exposures and the onset of SUD and mental health disorders cannot be definitively established, and findings should be interpreted as associative rather than causal. Parental death may also increase children’s exposure to subsequent ACEs, such that elevated risk observed in adulthood may reflect downstream adversity following bereavement rather than the bereavement event itself. Because childhood exposures were assessed retrospectively, findings may also be subject to recall bias. The reporting of parental substance use problems and parental/OA suicide may be influenced by social desirability or stigma-related underreporting. Additionally, parental/OA suicide was assessed as suicide by a parent or another adult living in the household during childhood, and it was not possible to distinguish parental suicide from suicide by other household adults. This measurement limitation may have introduced heterogeneity in the exposure and could have influenced the strength and interpretation of observed associations. Additionally, we were unable to determine specific causes of parental death; future research should examine whether deaths from stigmatized causes (e.g., drug overdose) place bereaved children at greater risk for SUD and mental health disorder outcomes than other causes of parental death. Furthermore, parental death was assessed regardless of cause, and parental/OA suicide represents a specific cause of death; as such, these exposures are not entirely distinct, which may complicate interpretation of their independent associations and introduce overlap in classification. Moreover, the DSM-5’s other drug use disorder outcome combined multiple substances with heterogeneous risk profiles, including differences in overdose risk and infectious disease transmission, which limits substance-specific interpretation of findings. Future research should disaggregate specific drug classes to better inform prevention and harm reduction strategies. Additionally, parental substance use may confound associations between bereavement and offspring outcomes by increasing both the likelihood of premature parental mortality and shared genetic or environmental risk for substance use and mental health disorders in offspring, potentially biasing estimates of bereavement effects. Additionally, NESARC-III data were collected in 2012–2013, largely preceding the widespread proliferation of fentanyl in the U.S. drug supply, and patterns of parental substance use, overdose mortality, and related risks for children may differ in more recent cohorts. Although the NESARC-III survey weights adjust for nonresponse, the response rate of 60.1% raises the possibility of residual nonresponse bias. Future research would also benefit from prospective cohort designs that can more precisely establish temporal ordering of parental death, other adverse childhood experiences, and psychiatric disorder onset. In addition, mediation analyses examining the timing and types of ACEs, as well as genetically-informed or family-based designs (e.g., sibling-comparison studies), may help disentangle potential familial and genetic confounding in these associations. Parental substance use may also contribute to both premature parental mortality and inherited vulnerability to substance use and mental health disorders in offspring, raising the possibility that shared genetic liability, rather than parental death itself, may partially account for observed associations. Finally, detailed measures relating grief reactions to parental death and parental substance use, including maladaptive grief reactions, were not available. This additional insight into the mechanisms of SUD and mental health disorder risk is an important area for future research.

In conclusion, our study highlights the potentially deleterious influence of cumulative trauma on the health of parentally-bereaved individuals. Healthcare professionals working with parentally-bereaved children should be aware that SUDs, suicidality, and mental health disorder risk is greater for children who experience both parental death and parental substance-related problems. Any parental death is a significant event for children, and this study indicates parental substance use history and parental suicide may be associated with elevated risk for suicidality and DSM-5 substance use and mental health disorders and warrants more research attention. Individuals who experienced parental death may benefit from the implementation of comprehensive screening, including for suicidality, SUDs, and mental health disorders. These findings also underscore the need for tailored bereavement interventions that use a trauma-informed approach and for such services to be readily accessible in communities with high rates of parental death and substance use problems.

## Supplementary Information

Below is the link to the electronic supplementary material.

Supplementary Material 1

## Data Availability

Researchers can apply for access to data from the NESARC-III study through the National Institute on Alcohol Abuse and Alcoholism (https://www.niaaa.nih.gov/research/nesarc-iii).
