# Supplementary material for: Parental death and parental substance use problems during childhood and the risk of DSM-5 substance use, suicidality and mental health disorders
Source: Soc Psychiatry Psychiatr Epidemiol. 2026 Mar 13;61(9):1781–93. doi: 10.1007/s00127-026-03076-6 (PMC13384750; doi:10.1007/s00127-026-03076-6)
Supplement: Supplementary file 1 [file 127_2026_3076_MOESM1_ESM.docx]

Supplemental Table 1. Associations of parental death, parental substance use problems and parental/other adult (OA) suicide with lifetime substance use disorders, suicide attempt, and mental health disorders

|  | **Lifetime AUD** | **Lifetime DUD** | **Lifetime**  **TUD** | **Lifetime**  **Suicide Attempt** | **Lifetime Mood Disorder** | **Lifetime Anxiety Disorder** | **Lifetime PTSD** |
| --- | --- | --- | --- | --- | --- | --- | --- |
| **Parental death and parental substance use problem (Models 1-7)** | aOR  (95% CI) | aOR  (95% CI) | aOR  (95% CI) | aOR  (95% CI) | aOR  (95% CI) | aOR  (95% CI) | aOR  (95% CI) |
| Neither | 0.39  (0.33, 0.45)* | 0.29  (0.23, 0.36)* | 0.35  (0.30, 0.40)* | 0.32  (0.24, 0.41)* | 0.49  (0.41, 0.58)* | 0.56  (0.46, 0.69)* | 0.41  (0.31, 0.54)* |
| Parental death + NO parental substance use problem | 0.45  (0.36, 0.55)* | 0.37  (0.26, 0.53)* | 0.46  (0.38, 0.55)* | 0.39  (0.28, 0.55)* | 0.54  (0.43, 0.69)* | 0.60  (0.45, 0.79)* | 0.56  (0.38, 0.82)* |
| NO parental death + parental substance use problem | 0.86  (0.73, 1.00) | 0.80  (0.64, 1.00) | 0.72  (0.62, 0.84)* | 0.78  (0.60, 1.02) | 0.94  (0.79, 1.12) | 0.97  (0.80, 1.18) | 0.90  (0.68, 1.20) |
| Parental death + substance use problem + NO parental/OA suicide | REF | REF | REF | REF | REF | REF | REF |
| Parental death + substance use problem + parental/OA suicide | 1.26  (0.68, 2.31) | 1.38  (0.66, 2.87) | 1.44  (0.76, 2.74) | 3.25  (1.67, 6.33)* | 1.87  (0.92, 3.79) | 3.16  (1.52, 6.58)* | 2.55  (1.08, 6.06)* |

Note: aOR=adjusted odds ratio. All models adjusted for sex, age, race/ethnicity, education, sexual identity, region, and urbanicity.

*p<0.05
